# 

**Published:** 1877-07

**Authors:** 


*May 1st*, 1877.

The first Annual Meeting of the American Dermatological Association will be held at Niagara Falls on the fourth day of September next.

"The titles of all papars to be read at any annual session shall be forwarded to the secretary, not later than one month before the first day of the session."

Respectfully yours,

Officers

- JAMES C. WHITE, M. D., PRESIDENT,

- LOUIS A. DUHRING, M. D., ROB'T W. TAYLOR, M. D., VICE-PRESIDENTS.

- L. DUNCAN BULKLEY, M. D., SECRETARY,

- JAS. NEVINS HYDE, M. D., TREASURER.

:o:

### The Popular Science Monthly.

### Books and Pamphlets Received.

---

Ziemssen's Cyclopædia. Volume XV. Diseases of the Kidney. New York : Wm. Wood & Co., 1877. Buffalo : H. Mattison.

How to Use the Ophthalmoscope. By Edgar A. Browne. Philadelphia : Henry C. Lea, 1877. Buffalo : T. H. Butler.

Transactions of the American Gynæcological Society. Boston: H. O. Houghton & Co., 1877. Buffalo: Peter Paul & Bro.

The Question of Rest for Women During Mensturation by Mary Putnam Jacobi, M. D. New York: G. P. Putnam's Sons, 1877. Buffalo: Peter Paul & Bro.

Hand Book for Hospital Visitors. New York: G. P. Putnam's Sons, 1877. Buffalo: Peter Paul & Bro.

Micro Photographs in Histology. Vol. I, No. 9. By Carl Seiler, M. D.

The Prophylactic Treatment of Placenta Praevia. By T. Gaillard Thomas, M. D.

The Speciality of Diseases of Women. By Clifton E. Wing, M. D., Boston.

The Woodruff Scientific Expedition around the world, 1877–9.

Thacheotomy in Diphtheria. By J. H. Pooley, M. D.

Report of Eye and Ear Department of Philadelphia Dispensary, 1876.

Association of the Alumni, of the Albany Medical College, 1877.

Report of the North-Eastern Dispensary in the City of New York, year, 1876.

Report of the Resident Physician of Brigham Hall, for the year 1876.

Syphilis and Chancroid. By P. H. Bailhache, Surgeon U. S. Hospital Service.

Two cases of Oesophagotomy. By LeRoy McLean, M. D., Surgeon to Troy Hospital.

The Relation of Ancient Medicine to Gynæcology, By Edward W. Jenks, M. D. Reprint from Detroit Medical Journal, 1877.

### INDEX.

**A**

A Bloodless Operation, .................. 192
Abuse and Use of Bromides, ............ 303
Abstract of the Proceedings of the Buffalo Medical Association, .....3, 86, 169, 258
A Case of Hydrophobia, ................. 231
A Case of Phthisis, with Vomica Communicating with Gaseous Tumor on Anterior Part of Chest, ............... 111
A Case of Uretro-plasty, ................ 15
Accidental Loss of Drainage Tube in the Pleural Cavity, ...................... 226
Action of Gelsiminum Sempervirens, .... 314
Address to the Graduating Class, Buffalo Medical College, Feb. 20, 1877, By Prof. E. V. Stoddard, M. D. ................ 203
Advanced Thinkers, ..................... 191
Air, Relation of, to the House we live in, 343
Albumen in the Treatment of Pnlmonary Consumption, By E. L. Shurly, M. D. ....... 283, 327
Albuminuria, Management of, ........... 22
Albuminuria, Production of, ............ 348
Anæsthesia, Bonwill's Method of ........ 17
Anæsthesia, by Ether in Young Subjects, Symptoms resulting from, ............. 274
Anæsthesia by the Injection of Chloral into the Veins, ...................... 230
Aneurism, Popliteal, By Prof. Julius F. Miner, M. D. ......................... 1
Aneurism, Safe and Rapid Cure for, ..... 348
A New Poison, .......................... 181
Anti-zymotic treatment of Diphtheria, .. 191
Aspiration in the Treatment of Hernia, . 229

**B**

Barnes, E. R., M. D., The Hip Joint, .217, 292
Blackham, Geo. E., M. D., Notes on Microscopy, .............................. 9
Blue Glass, ............................ 271
Books and Pamphlets Received, .......... 39, 122, 162, 202, 241, 282, 322, 355
Bonwill's Method of Anæsthesia, ........ 17
Bradnack, F., M. D., The Therapeutics of Periodical Cephalgia, ................ 243
Brain, Macro-Microscopical Sections of, . 178
Bromide of Potassium as a Caustic, ..... 314
Bromides, The Use and Abuse of, ....... 303
Brush, Edward N., M. D., Malignant Disease of the Tonsil, Removal by Galvano-Cautery, ........................ 46
Brush, E. N., M. D., Notes on Obstetrics and Gynæcology, ..................... 12
Brush, E. N., M. D., Sarcoma of the Dura Mater, ............................. 126
Buffalo Medical Association, Abstract of the Proceedings of, ...........3, 86, 169, 258
Buffalo Medical Club, .................. 299

**C**

Case of Membraneous Croup, New Method of Treatment, ..................... 345
Cephalgia, Periodical, The Therapeutics of, By F. Bradnack, M. D., ............ 243
Chloral, Anæsthesia by Injection of, into the Veins, .......................... 230
Chloral, Relief From Pain by the Outward Application of, ................. 313
Cities, Health of, ...................... 348
Clinical Conversations on Diseases of the Skin, .............................. 154
Clinical Notes, By Julius A. Post, M. D., 85
Compulsory Medication of Prostitutes by the State, ......................... 177
Concerning "Blue Glass," .............. 271
Consciousness, Effects of Loss of, Upon Memory of Preceding Events, ......... 27
Correspondence, ....................116, 131
Croup and Diphtheria, .................. 172
Croup and Diphtheria, Some Practical Observations on the Differential Diagnosis of, ............................ 150

**D**

Diptheria, Anti-zymotic Treatment of, .. 191
Disenfecting Oven in Mercy Hospital, ... 346
Duodenum, Laceration of, Post Mortem Examination, By Prof. E. V. Stoddard, M. D. .............................. 337
Drainage Tube, Accidental loss of, in the Pleural Cavity, ..................... 226

**E**

Editorial.
American Dermatological Society, ... 351
American Gynæcological Society, ... 318
American Medical Association, ....... 275
American Medical Colleges, ........ 318
Annual Commencement Buffalo Medical College, ........................ 194
Back Numbers, ...................... 198
Bergman vs. Volker, ............... 158
Bogus Diplomas, .................... 180
Buffalo Medical and Surgical Journal, 193
Canadian Medical Association, ...... 36
Changes in Drug Trade, ............. 235
Convention of the Kentucky State Medical Society, .................. 317
Death of Dr. Chas. Winnie, .......... 315
Dialysed Iron, ...................... 351
International Medical Congress, ..... 81
Malpractice and Medical Testimony, 117
Medical and Surgical Results of the War, ............................ 34
Meeting of the Alumni Association Buffalo Medical College, ........160, 195
Meeting of the New York State Medical Society, ...................315, 317
Meeting of the Medical Societies, .... 349
One Thousand Dollars Reward, ..... 276
Progress or Something New, ......... 274
The "Bergman vs. Volker" Case Settled, .......................... 234
The Popular Science Monthly, ....... 351

EDITORIAL CONTINUED.
The Situation, 233
United States Pharmacopœia, 197
Valuable New Books, 315
Volume XVI., 349
Womens Hospital, 350
Effects of Loss of Consciousness Upon Memory of Preceding Events, 27
Epilepsy, The Rational Treatment of, By Reuben A. Vance, M. D., 123
Esmarch's Bandage and its Substitutes, 25
Excision of the Rectum for Carcinoma, 179

**F**

Fascia Lata, Its use in Standing. etc., 144
Feasibility of Extirpating the Thyroid Gland in Some Cases of Disease, With Report of Case. By Prof. J. F. Miner, M. D. 323
Fordyce, A. B., M. D. Puerperal Convulsions, Cases with Symptoms and Treatment, 163

**H**

Health Boards, State, 274
Health of Cities, 348
Hernia, Aspiration in the Treatment of, 229
Hip Joint, The, By E. R. Barnes, M. D., 217, 292
Hydrophobia, A Case of, 231
Hydrophobia or Tetanus, 228

**L**

Laceration of the Duodenum, Post Mortem Examination, By Prof. E. V. Stoddard, M. D., 337
Lost Art in Surgery, 346

**I**

International Medical Congress, 48

**M**

Macro-Microscopical Sections of the Brain, 178
Malignant Disease of the Tonsil, Removal by the Galvano-Cautery, By Edward N. Brush, M. D. 46
Malpractice, Suit for, Involving the Surgery of the Hip Joint, 89
Management of Albuminuria, 22
Medical Congress, The International, 48
Miner, J. F., M. D., Feasibility of Extirpating the Thyroid Gland in Some Cases of Disease, With Report of Case, 323
Miner, Prof. Julius F., M. D., Popliteal Aneurism, etc., 1
Miner, Prof, Julius F., M. D., Suggestions as to a New Method of Closing Recto-Vaginal Fistula, 83
Moore, E. M., Jr., M. D., Report of a Case of Sub-luxation of the Inferior Maxilla Backwards, 255

**N**

New Method of Treating Urethral Stricture, 190
New York State Medical Society, 338
Norfolk District Medical Society, 310
Notes on Local Treatment of Certain Skin Diseases, 19
Notes on Microscopy, By Geo. E. Blackham, M. D. 9
Notes on Obstetrics and Gynæcology, By E. N. Brush, M. D. 12

**O**

Opium Antidotes Exposed, 109, 308
Ovarian Tumor, Spontaneous Cure of, 18
Ovariotomy, The Treatment of the Pedicle In, By F. W. Strange, M. R. C. S. 41
Ovariotomy, Vaginal, 147

**P**

Phthisis, A Case of, with Vomica, Communicating with Gaseous Tumor on Anterior Part of Chest, 111
Plants, Sending to Sleep, 190
Poison, A New, 181
Popliteal Aneurism, Tumor Exposed, Contents Evacuated and Vessels Ligated, By Prof. J. F. Miner, M. D. 1
Post, Julius A., M. D., Clinical Notes, 85
Prostitutes, Compulsory Medication by the State, 177
Public Health Questions in the United States, 185
Puesperal Convulsions, Cases with Symptoms and Treatment, By A. B. Fordyce, M. D. 163
Pupil, The Indications offered by the, 182

**R**

Recto-Vaginal Fistula, Suggestions as to a New Method of Closing, By Prof. J. F. Miner, M. D. 83
Rectum, Excision of, for Carcinoma, 179
Relation of Air to the House we live in, 343
Relief of Pain by the Outward Application of Hydrate of Chloral, 313
Remarks on the Enucleation of Uterine Fibroids, 104
Report of a Case of Subluxation of the Inferior Maxilla backwards, By E. M. Moore, Jr., M. D. 255
REVIEWS.
A Century of American Medicine, 1776-1876. By Edward H. Clarke, M. D., Henry Bigelow. M. D., Samuel D. Gross, M. D., T. Gaillard Thomas, M. D., and J. S. Billings, M. D. 200
A Course of Practical Histology. By E. A. Schæfer, M. D. 354
A Directory for the Dissection of the Human Body. By John Cleland, M. D., F. R. S. 354
A Manual of Percussion and Auscultation. By Austin Flint, M. D. 120
Annual Report of the Supervising Surgeon Gen'l. U. S. Marine Hospital Service, 352
A Practical Treatise on the Diseases, Injuries and Malformations of the Urinary Bladder, the Prostrate Gland and the Urethra. By Samuel D. Gross, M. D., LL. D., D. C. L., Oxon, etc. Edited By S. W. Gross, A. M., M. D. 281
A Practical Treatise on the Diseases of the Eye. By Robert Brudenell Carter, F. R. C. S. 319
A Series of American Clinical Lectures. Edited by E. C. Seguin, M. D. 236
Atlas of Skin Diseases. By Louis A. Duhring, M. D. 37
A Treatise on Hernia, With a Process for its Radical Cure, etc. By Greensville Dowell, M. D. 200
A Treatise on Surgery, its Principles and Practice. By T. Holmes, M. A., Cantab 198

REVIEWS CONTINUED.
A Treatise on the Science and Practice of Midwifery. By W. S. Playfair, M. D., F. R. C. P. .......... 121
Chemical and Microscopical Analysis of the Urine in Health and Disease. By Geo. B. Fowler, M. D. .......... 320
Chirurgie Antiseptique Principes Modes D applications et Resultants Pansament De Lister Par Le Dr. Just Lucas-Championniere, .......... 120
Contributions to Reparative Surgery. By Gurdon Buck, M. D. .......... 353
Cyclopædia of the Practice of Medicine. By Dr. H. VonZiemssen, .... 162, 199, 320
Healthy Skin; A Popular Treatise on the Skin and Hair. By Erasmus Wilson, F. R. S., F. R. C. S. .......... 278
Inhalation in the Treatment of Disease, Its Therapeutics and Practice. By J. Solis Cohen, M. D. .......... 121
Micro-Photographs in Histology. Normal and Pathological. By Carl Seiler, M. D., in Conjunction with J. Gibbons Hunt, M. D., and Joseph G. Richardson, M. D. .......... 118
Myelitis of the Anterior Horns. By E. C. Seguin, M. D. .......... 352
Specimen Fasciculus of a Catalogue of the National Medical Library, .. 38
Spiritualism and Allied Causes and Conditions of Nervous Derangement. By William H. Hammond, M. D. .......... 241
Studies, Chiefly Clinical, in the Non-Emetic use of Ipecacuanha. By Alfred Woodhull, M. D. .......... 201
The Electric Bath, Its Medical Uses, Effects and Appliance. By Geo. A. Schweig, M. D. .......... 239
The Microscope, a Manual of Microscopy, etc. By J. H. Wyeth, A. M., M. D. .......... 355
Theory and Practice of Medicine. By John Syer Bristowe, M. D., Lond, F. R. C. P. .......... 322
The Practitioner's Hand-Book of Treatment. Ry J. M. Fothergill, M. D., M. R. C. P. Lond. .......... 352
The Student's Guide to the Practice of Midwifery. By D. Lloyd Roberts, M. D., etc .......... 39
The Tonic Treatment of Syphilis. By E. L. Keyes, A. M., M. D. .......... 280
The Transactions of the American Medical Association at its Twenty-Seventh Annual Meeting, .......... 277
Transactions of the College of Physicians of Philadelphia. .......... 161
Rheumatism, Acute, Salicylic Acid in, .. 184
Rheumatism, Salicylate of Soda in, ..... 139

**S**

Safe and Rapid Cure for Aneurism, ..... 348
Salicylate of Soda in Rheumatism, ...... 139
Salicylic Acid, .......... 272
Salicylic Acid in Acute Rheumatism, .... 184
Sarcoma of the Dura Mater, By Edward N. Brush, M. D. .......... 126
Sending Plants to sleep, .......... 190
Shortening of the Lower Limb after Fracture of the Femur, .......... 268
Shurly, E. L., M. D., Albumen in the Treatment of Pulmonary Consumption 283, 327
Skin Diseases, Clinical Conversations on, 154
Skin Diseases, Notes on Local Treatment of, .......... 19
Some Practical Observations on the Differential Diagnosis of Croup and Diphtheria. .......... 150
Spontaneous Cure of Ovarian Tumor, ... 18
Surgery, Lost Art in, .......... 346
State Boards of Health, .......... 274
Stoddard, Prof. E. V., M. D., Address to the Graduating Class, Buffalo Medical College, Feby. 20, 1877. .......... 203
Stoddard, Prof. E. V., M. D., Laceration of the Duodenum, Post Mortem Examination, .......... 337
Strange, F. W., M. R. C. S., The Treatment of the Pedicle in Ovariotomy, ... 41
Suit for Malpractice, Involving the Surgery of the Hip Joint, .......... 89
Suggestions as to a New Method of Closing Recto-Vaginal Fistula, By Prof. Julius F. Miner, M. D. .......... 83
Sulphuric Acid in Necrosis, .......... 24
Symptoms Resulting from Anæsthesia by Ether in Young Subjects, .......... 274

**T**

Tetanus or Hydrophobia, .......... 228
The Fascia Lata, Its Use in Standing, at Rest, etc., .......... 144
The First Telegraph, .......... 180
The Indications offered by the Pupil, .... 182
Therapeutic Action of Carbolized Camphor, .......... 192
The Production of Albuminuria, .......... 348
The Rational Treatment of Epilepsy, By Reuben A. Vance, M. D., .......... 123
The Therapeutics of Periodical Cephalgia, By F. Bradnack, M. D. .......... 243
The Treatment of the Pedicle in Ovariotomy, By F. W. Strange, M. R. C. S. ... 41
Thyroid Gland, The Feasibility of Extirpating the, in some Cases of Disease, By Prof. J. F. Miner, M. D. .......... 323
Tonsil, Malignant Disease of, Removal by Galvano Cautery, By Edward N. Brush, M. D. .......... 46

**U**

Urethral Stricture, New Method of Treating, .......... 190
Uretro-plasty, A Case of, .......... 15
Use of Aromatic Sulphuric Acid in Necrosis, .......... 24
Uterine Fibroids, Remarks on the Enucleation of, .......... 104

**V**

Vaginal Ovariotomy, .......... 147
Vance, Reuben A., M. D., The Rational Treatment of Epilepsy, .......... 123
Venereal Ulcer or Chancroid, .......... 262

